# Beef, Casein, and Soy Proteins Differentially Affect Lipid Metabolism, Triglycerides Accumulation and Gut Microbiota of High-Fat Diet-Fed C57BL/6J Mice

**DOI:** 10.3389/fmicb.2018.02200

**Published:** 2018-09-24

**Authors:** Muhammad Umair Ijaz, Muhammad Ijaz Ahmed, Xiaoyou Zou, Muzahir Hussain, Min Zhang, Fan Zhao, Xinglian Xu, Guanghong Zhou, Chunbao Li

**Affiliations:** ^1^Key Laboratory of Meat Processing and Quality Control, MOE, Jiangsu Collaborative Innovation Center of Meat Production, Processing and Quality Control, Nanjing Agricultural University, Nanjing, China; ^2^Key Laboratory of Meat Processing, MOA, Jiangsu Collaborative Innovation Center of Meat Production, Processing and Quality Control, Nanjing Agricultural University Nanjing, China

**Keywords:** dietary proteins, gut microbiota, lipid metabolism, triglyceride accumulation, diet induced obesity

## Abstract

Consumption of dietary protein at recommended levels is considered a potential strategy to promote satiety and weight management, but how protein from different dietary sources effect the obesity development, lipid metabolism, and gut microbiota is not known. This study focused on the effects of beef, casein, and soy protein diet on lipid metabolism, triglycerides accumulation, and microbial diversity in colon of C57BL/6J mice, which were given either low-fat diets (LFD, 12% Kcal) or high-fat diets (HFD, 60% Kcal) for 12 weeks. Body and liver weight increased significantly in mice fed a beef protein HFD (HFB), whereas reduced cumulative energy intake was seen in a soy protein HFD (HFS) group. HFB-fed mice showed signs of impaired glucose metabolism and insulin resistance along with a significant elevation in the concentration of triglycerides, LDL-cholesterol, total cholesterol, IL1β, TNF-α, IL-6, and leptin in serum. HFB also enhanced lipid accumulation in liver with increased activity of genes important for lipogenesis and hepatic cholesterol metabolism. A 16S rRNA gene sequencing indicated that HFD, regardless of proteins, significantly enhanced the ratio of *Firmicutes* to *Bacteroidetes* in colonic microbiota. However, HFB not only reduced the abundance of *Akkermansia*, compared with LFD independent of proteins, but also decreased the abundance of butyrate-producing bacteria such as *Anaerotruncus*, *Butyricicoccus*, and *Lactobacillus* (*P* < 0.05) compared with HFS and HFC. In conclusion, consumption of HFB does not only affect the gut microbiota composition but also increases the problems related to metabolic syndromes like dyslipidemia, hypercholesterolemia, and triglycerides accumulation in liver, which lead to systemic inflammation and its associated comorbidities, for example, impaired glucose metabolism and insulin resistance.

## Introduction

In past decades, an unprecedented increase has been seen in metabolic syndromes including obesity, diabetes, hyperlipidemia, hyperinsulinemia, and cardiovascular disease (Shang et al., 2017). Unfavorable patterns of food intake may cause dysfunctions in lipid metabolism and metabolic syndromes because it has been established that accumulation of lipid and triglycerides in liver consequently results in fatty liver disease, hepatic steatosis, and even diabetes (Maeda et al., 2015; Liisberg et al., 2016).

Metabolic syndromes is a constellation of disorders including hyperlipidemia, hyperinsulinemia, hypertension, cardiovascular diseases, and type 2 diabetes (Després et al., 2006; Eckel et al., 2010). Consumption of high caloric foods and sedentary lifestyle are the two main reasons for metabolic syndromes (Strasser, 2013).

In recent years, modulation of dietary protein intake has been considered a potential strategy for obesity alleviation and weight management. Increasing protein content in diets has been suggested as a potent way to deal diet-induced obesity (Wycherley et al., 2012; Ayala et al., 2016). High-protein in diet can increase satiating effect and thermogenesis (Weigle et al., 2005). Diets rich in taurine, lysine, arginine, and glycine are reported to have an anti-inflammatory effect (Dort et al., 2013).

Gut microbiome plays a major role in energy harvesting and obesity development. Dietary intervention is a promising way by manipulating the composition of gut microbiota and its potential effects on host metabolism (Turnbaugh et al., 2009).

Previous studies have revealed that intestinal microbiota has strong associations with dyslipidemia, insulin resistance, and glucose metabolism (Everard et al., 2013). *Akkermansia*, *Christensenellaceae*, and *Tenericutes* are reported to have the ability to increase lipid metabolism and high-density lipoproteins (Everard et al., 2011; Fu et al., 2015). *Akkermansia muciniphila* can also reduce gonadal fat mass gain and triglyceride in serum and liver (Org et al., 2015). Recently L-carnitine, a nutrient abundantly present in red meat, was found to elevate CVD risk by changing gut microbiota composition and increasing TMAO production in host (Koeth et al., 2013).

Considering these effects of diet on obesity, it is imperative to consider how intake of protein from different sources affects lipid metabolism, hypercholesterolemia, gut microbiota, and other metabolic markers associated with metabolic syndromes.

In this study, we investigated the effects of beef protein, casein, and soy protein in both lean and obese C57BL6/J mice [which were fed high-fat diet (HFD) to induce obesity] by examining lipid metabolism, triglyceride accumulation, insulin resistance, and colonic microbial diversity.

## Materials and Methods

### Animals and Diets

Seven weeks old C57BL/6J mice (Male, *n* = 60) from NBRI (Biomedical Research Institute) were kept in an animal center (SYXK < Jiangsu > 2011-0037) under specific pathogen free conditions. After 1 week of acclimatization, 30 mice were randomly assigned to a low-fat diet (LFD) **(**D12450J, 12% kcal from lard New Brunswick, United States) and the remaining 30 mice were subjected to a HFD **(**D12492, 60% kcal from lard, New Brunswick, United States) See **Supplementary Table S1**. For soy protein diet and beef protein diet, casein was replaced in both D12450J and D12492 by protein powders extracted from soy and beef. Mice were given *ad libitum* access to diets and clean water. Diet intake and body weight were recorded at a 2-week interval. Diets were changed every 3 days. All animal handling was done in compliance with the guidelines given by the Ethical Committee of Experimental Animal Center of Nanjing Agricultural University.

### Glucose and Insulin Tolerance Tests

For glucose tolerance test (GTT), mice were fasted for 6 h prior to testing, and then intraperitoneally injected with 2.0 g dextrose–glucose/kg lean mass. Blood samples were drawn from the tail vein before injection or at 15, 30, 60, 90, and 120 min after injection. Glucose concentrations were measured using a handheld glucometer (ACCU-CHEK, Roche, Shanghai, China).

For insulin tolerance test (ITT), animals were fasted for 6 h before testing and insulin (0.75 U/kg body weight) was injected intraperitoneally. Blood was drawn from the tail vein before injection or at 15, 30, and 60 min after injection. Glucose concentrations were measured by the handheld glucometer. Trapezoidal method was used to determine incremental area under the curve and decremental area under the curve for glucose and insulin, respectively.

### Collection of Samples

After 12 weeks, mice were sacrificed and blood was collected. To get serum, blood was left to stand for 50 min at room temperature, and then samples were centrifuged for 30 min at 12,000 ×*g* to pellet the blood cells. Serum was stored at -80°C. The content from colon was collected, snap frozen, and stored at -80°C for 16S rRNA sequencing. Liver samples were weighed, snap frozen, and then stored at -80°C.

### Biochemical and Cytokine Analyses

The aspartate aminotransferase (AST) and alanine aminotransferase (ALT) in serum were measured at 542 nm and 525 nm, respectively, by a microplate spectrophotometer (MD, United States) using a commercial kit (A042, Nanjing Jiancheng Bioengineering Inst.) following the manufacturer’s instructions. Triglycerides (TG), total cholesterol (TC), HDL-C, LDL-C, total bile acids (TBA), and glucose (Glu) levels in serum were measured using a Chemray 240 automatic chemistry analyzer (Rayto, Shenzhen, China). The TG concentration in liver was determined using an ELISA kit (Elabscience, No. E-EL-M2603c). The serum LBP level was determined using a commercial ELISA Kit (No. RGB-60178M, Beijing Rigor Bioscience Development, Ltd.). Concentrations of IL1β, TNF-α, IL-6, and leptin in serum were measured with a Bio-Plex kit (5827, Bio-Rad Laboratories, Inc.).

### Histochemical Analyses

Liver samples were fixed in 10% formalin and embedded in paraffin. The paraffined tissues were sectioned to 6 μm thickness and stained with H&E solution to analyze hepatic vacuolization. To further confirm the vacuolization and quantify lipid droplets, frozen liver samples were sectioned to 8 μm thickness and stained with 0.2% Oil Red O in 60% of isopropanol for 20 min, and then PBS was used to wash the liver sections three times. A regular light microscope was used for microscopic examination and images were analyzed using Image-Pro Plus for estimating lipid droplet percent in liver tissues.

### Gene Expression Analysis by Real-Time PCR

Total RNA from liver tissue was extracted using RNeasy mini kit (Takara, Code: 9767), and cDNA was prepared mixing 4 μL RNA and 2 μL 5× PrimeScript RT Master MIX (Takara, Code: RR036A) in RNase free water up to 10 μL. A QuantStudio 6 Flex Real-time PCR system was used for relative quantification of gene expression with SYBR green probe. Relative gene expression was analyzed with 2^-ΔΔCt^ method (Livak and Schmittgen, 2001). The mRNA levels in test samples were normalized relative to GAPDH and calculated as fold change (2^-ΔΔCt^) in which soybean group was set as a control. See **Supplementary Table S2** for target primers list (Sangon Biotech).

### 16S rRNA Gene Sequencing

#### Extraction of DNA From Colon Content and PCR Amplification

A TIANamp Stool DNA Kit (DP328) was used to collect microbial DNA from colonic content following manufacturer’s instructions. After DNA extraction, primers 515F 5′-barcode-GTGCCAGCMGCCGCGG-3′ and 806R 5′-GGACTACHVGGGTWTCTAAT-3′ were used to amplify the V4 region of bacterial 16S ribosomal RNA gene in which barcode was unique in each sample with an eight-base sequence. After amplification with PCR, amplicons were extracted from agarose gel (2%) and purification was done with the help of DNA gel extraction kit (Axygen Biosciences, Union City, CA, United States).

#### Library Construction and Data Processing

Purified PCR products were quantified using Qubit^®^3.0 (Life Technologies), and each 24 amplicons in samples were integrated with different barcodes. Illumina Pair-End library was created using pooled DNA product following procedures of Illumina’s genomic DNA library. The Illumina Miseq (Shanghai Biozeron Co., Ltd.) was used to make paired-end sequences (2 × 250) of the amplicon library as described in the standard protocols. Sequenced data were processed by demultiplexing raw fastq files and quality filtering it with QIIME software (version 1.9.0). The UPARSE (version 7.1) was employed to cluster OTUs at 97% similarity cutoff, and UCHIME was used to distinguish and eliminate chimeric sequences. The phylogenetic connection of 16S rRNA gene sequence was determined by RDP Classifier against SILVA (SSU123), data obtained after 16S rRNA was sequenced using a confidence level of 70% (Amato et al., 2013).

### Microbial Diversity Analyses

Mothur v.1.21.1 was used to identify alpha diversity indices, including Shannon diversity indices, Chao and Good’s coverage diversity indices (Schloss et al., 2009). UniFrac was used for beta diversity analysis (Lozupone et al., 2011) Vegan 2.0 package for package R.

### LEfSe Analysis

For identification of biomarkers for highly dimensional colonic bacteria, LEfSe (linear discriminant analysis effect size) analysis was done (Segata et al., 2011). Kruskal–Wallis sum-rank test was performed to examine the changes and dissimilarities among classes followed by LDA analysis to determine the size effect of each distinctively abundant taxa (Zhu et al., 2015).

### Statistical Analyses

Effects of diet on measured variables were evaluated by analysis of variance and Duncan’s multiple comparisons. Means were considered different at *P* < 0.05. Statistical analyses were done using GraphPad Prism (version 7, GraphPad Software INC., La Jolla, CA, United States).

## Results

### Growth Performance and Energy Intake

#### HFB Increased Energy Intake and Body Weight of Mice

**FIGURE 1:**
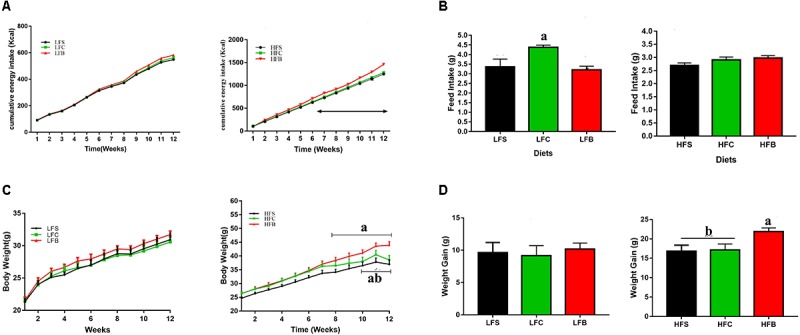
Feed intake and weight gain of mice in response to different diets. **(A)** Cumulative energy intake. Arrow indicates significant increase in energy intake of HFB-fed mice (*P* < 0.05). **(B)** Daily feed intake. **(C)** Growth curve. Arrow indicates significant difference (*P* < 0.05). **(D)** Total weight gain. The data represent group means (*n* = 10/group) ± SEM. Different letters denote statistical significance (*P* < 0.05).

Intake of HFB increased overall energy intake compared with HFS and HFC, and this difference was significant from the sixth week (**Figure 1A**). After 9 weeks feeding, mice fed HFB had significantly heavier body weight than those fed HFS and HFC (*P* < 0.05, **Figure 1C**). The body weight gain was significantly higher for HFB group (*P* < 0.05, **Figure 1D**).

In LFD groups, protein from different dietary sources had no significant effect on the cumulative energy intake and body weight gain in all groups during whole feeding trial, although mice fed casein had higher daily feed intake (*P* < 0.05, **Figure 1B**).

#### HFB Impaired Glucose Metabolism and Induced Mild Insulin Resistance

**FIGURE 2:**
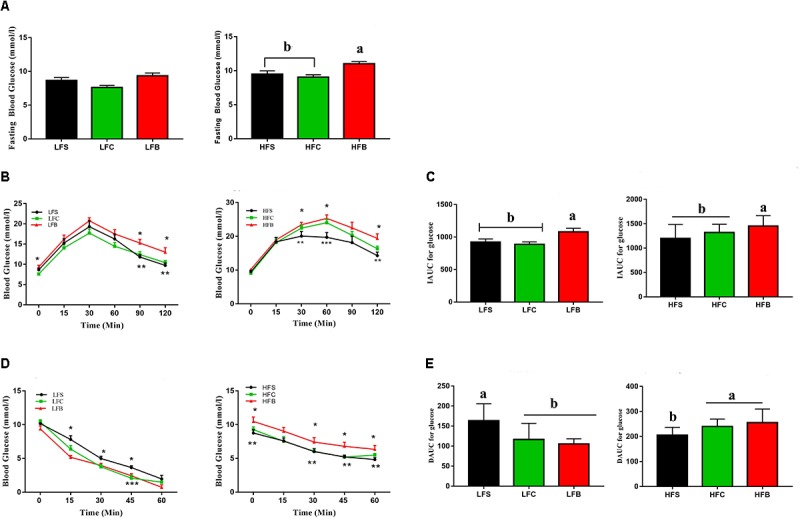
Glucose and insulin tolerance. **(A)** Feed-deprived (6 h) blood glucose levels before starting the oral glucose tolerance test (*n* = 10/group). **(B)** Glucose response curve after administration of 3-mg glucose/g lean mass by gavage (*n* = 10/group). **(C)** Incremental area under the curve (IAUC) (*n* = 10/group). **(D)** Glucose response curve after administration of 0.75 units insulin/kg lean mass, *n* = 10/per group except in LF where *n* = 8 at time point 0 and 15 min and *n* = 6, 7, and 8, respectively, for beef, soy and casein protein at 30 min (four mice were treated with glucose due to low blood glucose levels and data points after treatment were therefore not included). **(E)** Decremental area under the curve (DAUC), *n* = 10 for the high fat groups and *n* = 6 in low fat groups. The data represent group means ± SEM and different alphabetic letters and numerical asterisk (∗) denote statistical significance (*P* < 0.05).

To confirm if this increase in body mass also affect the glucose metabolism, we performed glucose tolerance test during 10th week. Mice fed HFB had higher fasting blood glucose concentration than those fed HFS and HFC (*P* < 0.05, **Figure 2A**). In addition, beef protein, regardless of HFD or LFD, increased glucose resistance compared with soy protein and casein (*P* = 0.05, **Figure 2B**), which was further confirmed by the differences observed in IAUC (*P* < 0.05, **Figure 2C**).

An insulin tolerance test indicated significant differences in insulin resistance among diet groups (*P* < 0.05, **Figures 2D,E**).

#### HFB Induced Hypercholesterolemia, Dyslipidemia, and Increased Triglyceride Concentration in Serum

**FIGURE 3:**
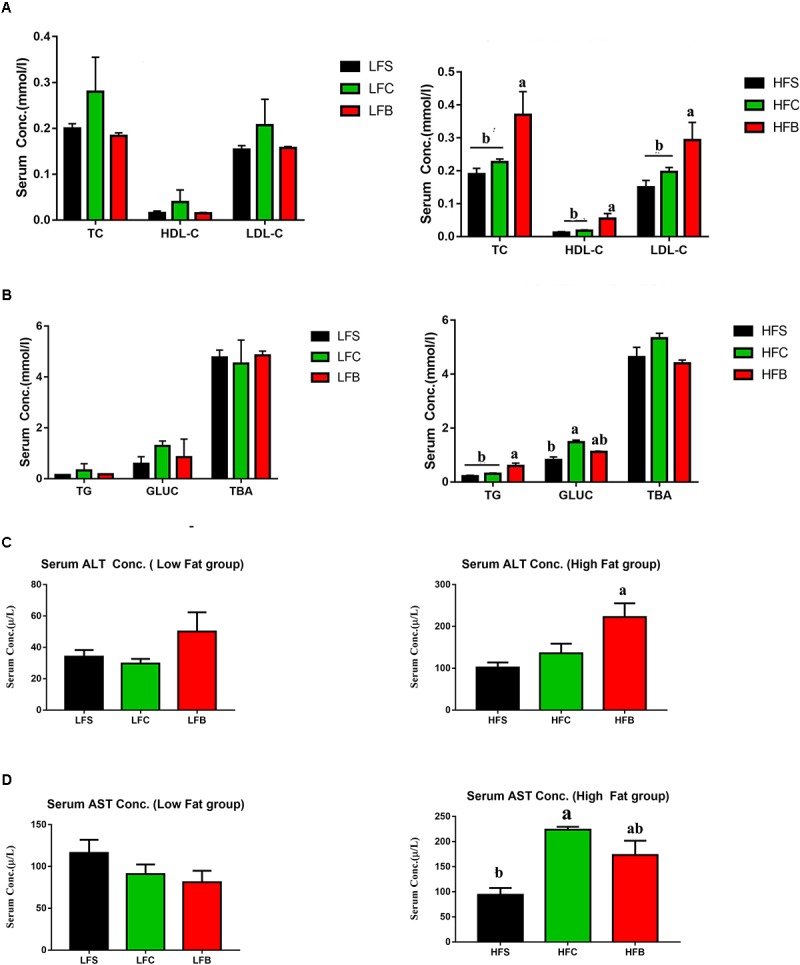
Serum lipid profile of C57BL/6 J male mice. **(A)** High-density lipoprotein cholesterol (HDL), low-density lipoprotein cholesterol (LDL), total cholesterol (TC); **(B)** triglycerides (TG), glucose (GLUC), total bile acid (TBA); **(C)** ALT; **(D)** AST. The data represent group means ± SEM and different letters denote statistical significance (*P* < 0.05).

Atherogenic dyslipidemia is referred as elevated level of triglycerides, LDL-C, and total cholesterol in serum (Grundy, 2018). HFB resulted in higher levels of TC, LDL-C, and HDL-C, which suggests that early signs of hypercholesterolemia occur in HFB group (*P* < 0.05, **Figure 3A**). At the same time, serum triglycerides (TG) and glucose (GLUC) were increased by HFB (*P* < 0.05, **Figure 3B**). No significant differences were seen in serum cholesterol and lipid profiles in LFD groups (*P* > 0.05, **Figures 3A,B**).

ALT has been suggested a potential serum marker for NAFLD, and much attention has been paid to its potential role in pathogenesis of metabolic syndromes (Schindhelm et al., 2014). Again, HFB increased ALT concentration in serum compared with HFS and HFC (*P* < 0.05, **Figure 3C**), but no significant differences in the above-mentioned variables among LFD groups were seen (*P* < 0.05, **Figures 3C,D**). Therefore, HFB increases the metabolic biomarkers linked to atherogenic dyslipidemia, hypercholesterolemia.

#### HFB Increased Systemic Inflammation and Endotoxin Concentration in Serum

**FIGURE 4:**
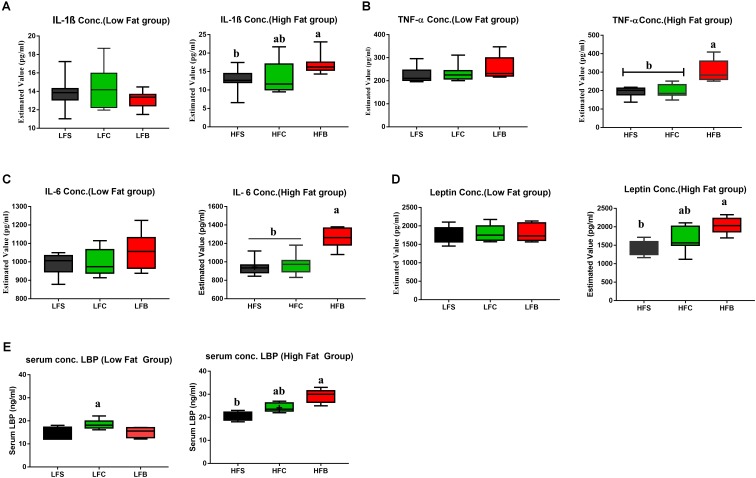
**(A–D)** Box-plots of serum inflammatory factors; **(E)** LBP. The data represent group means ± SEM and different letters denote statistical significance (*P* < 0.05).

Inflammation in liver is associated with fatty acid accumulation and cytochrome P450 inactivation that are regulated by several cytokines (Ronco et al., 2010). TNF-α may trigger the generation of secondary cytokines that increases neutrophil chemotaxis and leads to the inflammatory responses, which eventually results in necrosis and hepatosteatosis in liver (Ritter et al., 2003). HFB increased the concentrations of inflammatory cytokines in serum, including TNF-α, IL1β, IL-6, and leptin, suggesting that intake of beef protein lead to a significant increase in systemic inflammation (**Figures 4A–D**). No significant effects were seen on the concentrations of cytokines in low fat groups.

Concentration of lipopolysaccharide-binding (LBP) was measured in serum to analyze the antigen load of gut bacteria in mice. Compared with HFS and HFC, HFB induced higher level of LBP (*P* < 0.05, **Figure 4E**). For LFD groups, casein diet showed higher LBP concentration in serum. This indicates that the body gives different responses to dietary proteins in high-fat and LFD models.

#### HFB Increased Hepatic Lipogenesis and Cholesterol Metabolism

**FIGURE 5:**
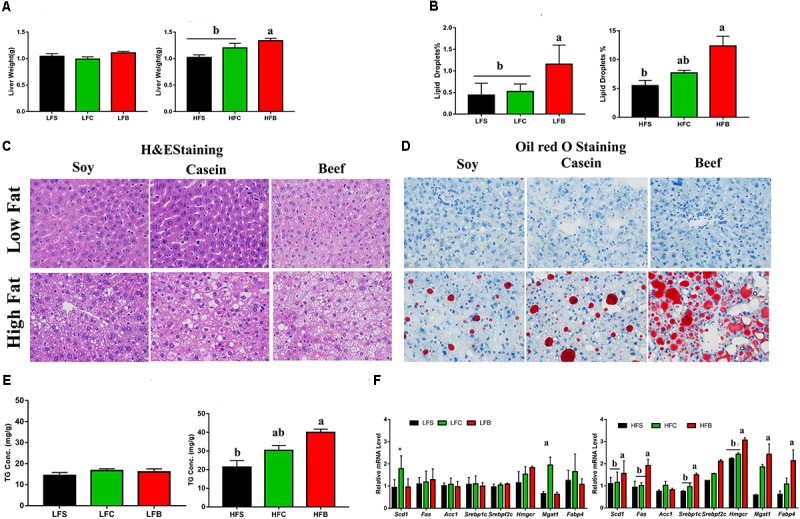
Lipid accumulation in liver: **(A)** Liver weight at the end of experiment; **(B)** lipid droplets percentage measured by Oil Red O staining; **(C)** Liver sections stained with H&E **(D)** or Oil Red O (original magnifications 400× for H&E and 200× for Oil red O staining); **(E)** triglycerides concentration in liver; **(F)** relative mRNA levels of genes involved in hepatic lipid and cholesterol metabolism. Each data point represents the mean ± SE and different letters and numerical asterisks denote statistical significance (*P* < 0.05). ^∗^*P* < 0.05, ^∗∗^*P* < 0.01 compared to internal control.

Consumption of HFB significantly increased liver weight (**Figure 5A**), which was accompanied with extensive hepatocyte vacuolization compared with HFS and HFC (**Figure 5C**). Oil Red O staining confirmed microstructural differences (**Figure 5D**) as percentage of lipid droplets in HFB group was significantly higher than other groups (**Figure 5B**). These results suggest that consumption of HFB increased hepatic lipid accumulation. It is known that an increase in IL-6 and leptin concentrations will enhance intrahepatic TG level (Perry et al., 2015). In this study, HFD, in particular to HFB, increased hepatic TG concentration (**Figure 5E**).

Considering obesity is partly linked with high lipid accumulation and high TG in liver (Fabbrini et al., 2010), which is regulated by *Hmgcr* (Min et al., 2012). RT-qPCR analysis indicated that consumption of HFB upregulated the expression of sterol regulatory element-binding protein-1 (*Srebp1c*), sterol regulatory element binding transcription factor 2 (*Srebpf2*), HMG-CoA reductase (*Hmgcr*), fatty acid synthase (*Fas*), stearoyl-CoA desaturase-1 (*Scd1*), monoacylglycerol *O*-acyltransferase 1 (*Mgat1*), and fatty acid binding protein 4 (*Fabp4*). No significant changes were seen in expression of the genes involved in lipid metabolism in LFD groups, except that Casein diet upregulated *Mgat1* expression (**Figure 5F**).

### Gut Microbiota

#### Richness and Diversity Analyses of Colonic Microbiota

Data of three samples from three mice in LFB group were removed due to fewer reads and death during insulin resistance test. A total of 1,172,273 usable raw reads were obtained from 26 samples with an average of 45,087 ± 7,326 reads per sample in HFD groups, corresponding with 1,139,234 usable reads from 25 samples in LFD groups and 45,569 ± 8,234 per sample (see **Supplementary Table S1**). Delineation of operational taxonomic units (OTU) was done at 97% similarity level. The total number of OTU was 11,929, with an average of 458 ± 78 per sample for HFD mice and 8,066 with an average of 322 ± 45 for LFD mice.

No significant differences (*P* > 0.05) were observed in both HFD and LFD groups in alpha diversity indices including Simpson index, Chao, Good’s coverage index, and Shannon index (**Supplementary Tables S3A,B**). Also rarefaction results at the OTU level revealed no significant differences between HFD and LFD groups (**Supplementary Figure S1**). These findings suggest that all samples had a great similarity in ecological diversity of gut microbiota in both HFD and LFD groups.

#### Overall Structure of Gut Microbiota

**FIGURE 6:**
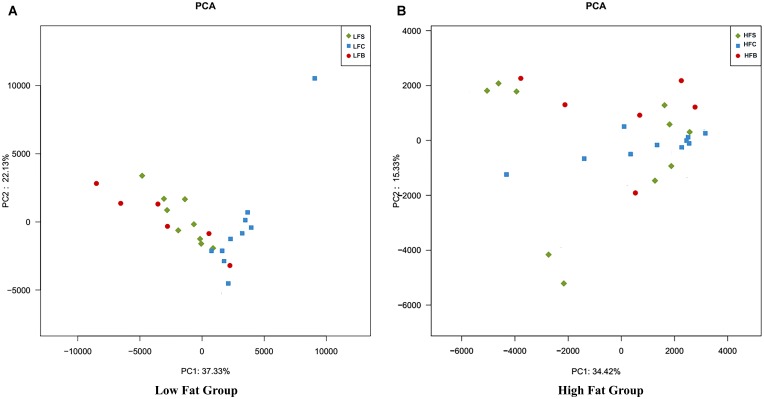
Principal component analysis of gut microbiota at the OTU level. Each point represents one sample. The numbers of animals for beef, casein, and soy protein groups are 6, 10, and 10 for both **(A)** high-fat diet and **(B)** low-fat diet.

To analyze and compare overall composition of colonic microbiota, multivariate analyses were performed on all colonic samples. PCA was done to see the changes in microbial composition of colonic contents among different groups. The first two components accounted for 59.46% variation in LFD groups and 49.75% variation in HFD groups (**Figures 6A,B**). PC1 mainly explains the diet-induced variations in microbial structure, while PC2 explains more intra-group variations.

In HFD groups, greater variation was seen among diet groups (**Figure 6**). Mice fed casein had a similar gut bacteria composition with small intra-group variation, but mice fed beef protein and soy protein exhibited greater intra-group variations. This suggests that gut microbiota had diverse response to beef protein (**Figure 6**). A similar behavior was seen in LFD groups (**Figure 6**).

**FIGURE 7:**
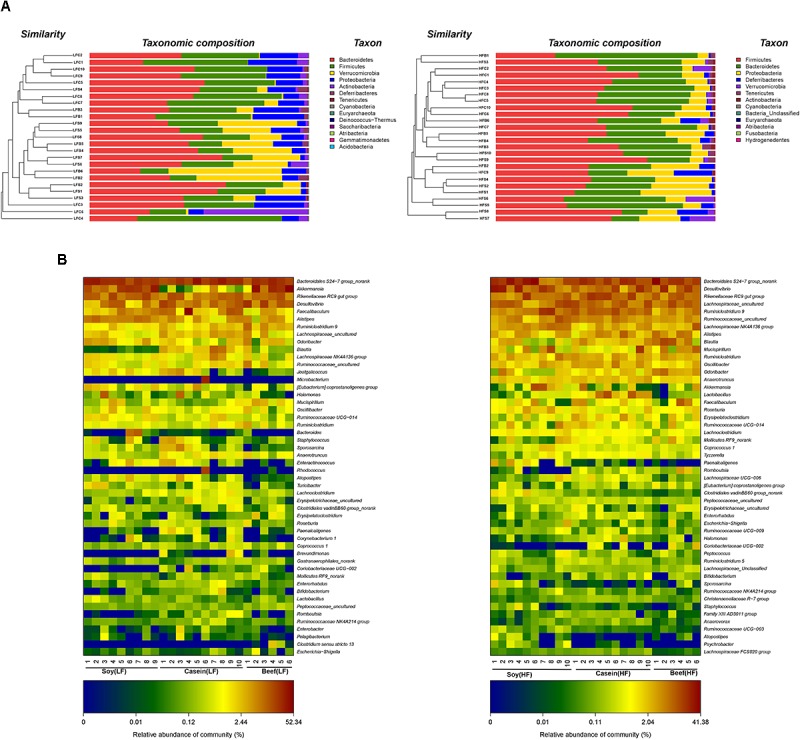
Clustering of gut microbiota. **(A)** Hierarchical clustering of colonic microbiota at the phylum level. Each line and bar represents the same sample. **(B)** Heatmap of gut microbiota at the genus level. Each column represents one sample and each row represents one genus. Note: The numbers of animals for low fat and high-fat diet groups are 25 and 26, respectively. With six in both high fat beef and low fat beef.

Hierarchical clustering analysis showed that HFD significantly increased *Firmicutes* to *Bacteroidetes* ratio compared to LFD (**Figure 7A**). *Firmicutes*, *Bacteroidetes*, and *Verrucomicrobia* were the most abundant phyla in both LFD and HFD groups.

Although HFD groups exhibited the signs of gut dysbiosis by showing an increase in the *Firmicutes* to *Bacteroidetes* ratio, but their relative abundances remained same in all HFD groups, that is, 46.75, 52.60, and 50.07% for *Firmicutes* and 30.06, 31.57, and 32.39% for *Bacteroidetes* in soy, casein, and beef protein diet groups, respectively. In addition, a great reduction in *Verrucomicrobia* abundance was observed in the HFB group compared with the LFB group and other HFD groups. The relative abundance of *Proteobacteria* in HFS group was higher than those in HFB and HFC groups. Differential sequences were matched with the top 50 genera (**Figure 7B**). In LFD groups, the most predominant genus was *Bacteroidales S24-7* (24.91%), which was followed by *Akkermansia* (12.68%), *Rikenellaceae RC9 gut group* (10.13%), *Desulfovibrio* (9.22%), *Faecalibaculum* (6.44%), *Alistipes* (5.13%), and *Ruminiclostridium 9* (2.49%). Consumption of HFD reduced the abundance of *Bacteroidales S24-7 group* (17.79%) compared with LFD, but it remained the most abundant genus. The other highly abundant genera include *Desulfovibrio* (11.45%), *Rikenellaceae RC9 gut group* (8.79%), *Lachnospiraceae uncultured* (8.29%), *Ruminiclostridium 9* (7.71%), *Ruminococcaceae uncultured* (4.71%), *Lachnospiraceae NK4A136 group* (3.80%), *Alistipes* (3.49%), *Blautia* (3.23%), and *Mucispirillum* (3.12%). HFD also increased the abundance of genera that are associated with diet induced obesity (Chang et al., 2015), including *Mucispirillum*, *Escherichia*, *Shigella*, *Mollicutes*, and *Oscillibacter* (Cani et al., 2007; Everard et al., 2011) and their relative abundances were highest in HFB group. Consumption of HFB drastically reduced the relative abundance of *Akkermansia* (up to -23%), but the diet induced an increase in relative abundance of *Anaerotruncus*, *bacteroides*, and *Blautia*. These genera are reported to have negative correlations with obesity (Million et al., 2012).

#### Linear Discriminant Analysis of Gut Microbiota

**FIGURE 8:**
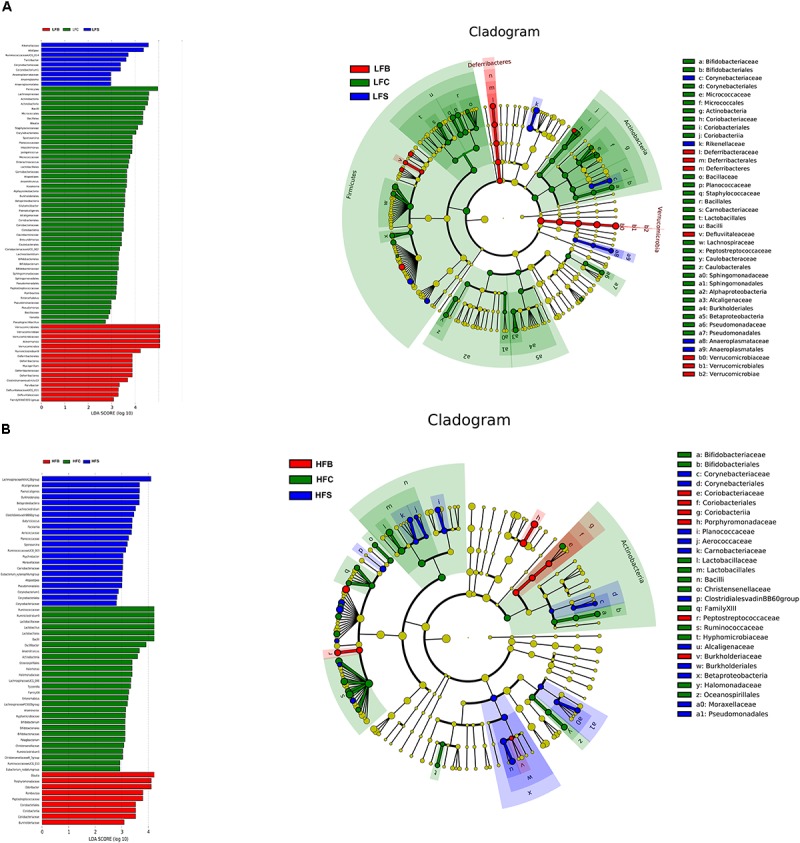
LEfSe analyses of gut microbiota data. Bacterial OTUs rich in the low-fat diet group **(A)** and the high-fat diet group **(B)**. The left histogram shows the LDA scores calculated for characteristics at the OTU level, while the right cladogram shows the relative abundance of OTUs. In the LEFse tree, different colors indicate different groups. Color shows an important microbial biomarker in the group and the biomarker name are listed in the upper right corner.

To identify the specific bacterial taxa associated with the dietary effects, we compared the colonic microbiota using LEfSe analysis. The results revealed 75 different OTUs among LFD groups and 59 different OTUs among HFD groups (**Figure 8**). In LFD groups, 50 OTUs were highly abundant in LFC group, with 16 in LFB group and 9 in LFS group. The LFS group had higher relative abundance of *Rikenellaceae* than LFB group (4.35% vs. 0.003%). *Akkermansia* was most abundant in LF beef protein group and least abundant in LFC group. LFB increased the relative abundances of *Mucispirillum*, *Deferribacteraceae*, *Desulfovibrionaceae*, and *Bacteroidaceae*. LFC group showed the highest relative abundances of *Firmicutes*, *Actinobacteria*, *Bacilli*, and *Lactobacillus*, but lower relative abundances of *Akkermansia*, *Deferribacters*, and *Ruminiclostridium.*

In HFD groups, 9 out of 59 OTUs were more highly abundant in HFS group, corresponding to 28 in HFC group and 9 in HFB group. *Lachnospiraceae NK4A136 group* was the most predominant bacteria in HFS group and the least abundant in the HFB group. The latter group had the highest relative abundances of *Blautia*, *Romboutsia*, and *Odoribacter*, whereas the HFC group was characterized by the highest relative abundances of *Ruminiclostridium 9*, *Lactobacillus*, *Anaerotruncus*, and *Actinobacteria.*

Taken together, dietary proteins, regardless of LFD and HFD, caused diverse changes in the microbial community in the colon. *Akkermansia* was the second most abundant genus in LFB group, but it was reduced by 23% in HFB group.

## Discussion

Obesity is characterized by an increased lipid accumulation in liver, which leads to hepatic steatosis and nonalcoholic fatty liver disease (NALFD) (Gonzalez-Periz et al., 2009). It is reported that high protein diets promote weight loss, satiety, and thermogenesis in human (Westerterp-Plantenga et al., 2012), but little is known on how the dietary proteins from different sources affect the microbial diversity, lipid metabolism, and triglycerides accumulation in both lean and obese conditions (Smith et al., 2015). Epidemiological studies suggest that consumption of protein from plant and dairy sources may protect against obesity, whereas increased consumption of meat, especially red meat, results in weight gain (Fogelholm et al., 2012; Mozaffarian, 2016). It is yet to be established how consumption of red meat protein affects the function and composition of the gut microflora in human. Composition of amino acids is also a key factor that determines the quality of dietary proteins (Millward et al., 2008). Different composition of amino acids in dietary proteins may have a great impact on body function and host metabolism (Tome, 2012).

In this study, mice in the LFD groups did not show significant changes in body weight gain, cholesterol metabolism, and hepatic lipid metabolism. However, LFC group showed relatively higher expression of *Mgat1* gene and triglycerides accumulation in liver.

In HFD groups, mice fed HFB showed significant phenotype changes associated with diet induced obesity, including impaired glucose and insulin metabolism, enhanced cumulative energy intake, increased level of LDL-C, TC, and TG concentration in serum. This increase in TG concentration in serum also induced secretion of cytokines, that is, leptin and IL-6, which are known to have an association with impaired glucose metabolism and insulin sensitivity (Morton and Schwartz, 2012). Therefore, consumption of HFB induced atherogenic dyslipidemia, systemic inflammation, and hypercholesterolemia.

The above differences among HFD groups can be further explained by an increased lipid accumulation in liver. Histological observations showed an increased hepatic vacuolization and lipid droplets in HFB group. As expected mice fed beef protein showed higher concentration of triglycerides in liver. Beef protein significantly increased lipogenesis by enhancing *Srebp1c* and its associated genes including *Acc1*, *Mgat1*, and *Fas*. Increased expression of *Srebp1c* has been reported to promote lipid accumulation in liver and fat tissue (Horton et al., 1998; Shimomura et al., 1999). Elevated expression of *Acc1* and *Mgat1* genes is directly linked to increased triglycerides accumulation and hepatic steatosis (Lee and Kim, 2017). And thus consumption of HFB increased lipid synthesis in liver and hepatic steatosis.

Gram-negative bacteria in gut are responsible for the production of lipopolysaccharide (LPS) (Fei and Zhao, 2013). Upon entering the circulation system, LPS upregulates LPB expression in liver, which further triggers gene expression of inflammatory cytokines including IL1β, TNFα, and IL-6 (Sun et al., 2010). Level of LBP in circulation is considered as a biomarker for anti-inflammatory response and antigen load to host (Zweigner et al., 2006). We found that HFB increased serum LBP level, which could be associated with changes observed in the composition of gut bacteria.

In recent years, an increased interest has been taken in associations among diet, gut microbes, and obesity. A study indicates that colonization of zebrafish gut with *Firmicutes* enhances epithelial absorption and energy balance of the host (Semova et al., 2012). Gut microbes can modulate the host metabolism and affect hypercholesterolemia, dyslipidemia, obesity, and diabetes (Tremaroli and Bäckhed, 2012; Karlsson et al., 2013; Ciciliot et al., 2018). It has been suggested that *Akkermansia, Christensenellaceae*, and *Tenericutes* have the ability to interact with lipid metabolism, body mass, and triglycerides accumulation in mice (Everard et al., 2011; Kitano et al., 2012).

We observed that consumption of HFB increased the relative abundances of *Mucispirillum, Escherichia*, *Shigella*, *Mollicutes*, and *Oscillibacter*, which are reported to positively correlate with obesity (Cani et al., 2007; Everard et al., 2011). On the other hand, HFB reduced the relative abundance of *Anaerotruncus*, *Bacteroides*, and *Blautia*, which have a negative correlation with obesity (Million et al., 2012). However, it is more interesting that HFB induced a greater reduction in the relative abundance of *Akkermansia* (up to -23%) compared with other diet groups.

Gut microbiota has the ability to help host harvest more calories from undigested ingredients of food by fermenting and producing SCFAs from the endogenous proteins (Kübeck et al., 2006). This symbiotic effect can describe the mechanism in which the soy protein group gained less body weight (Zhu et al., 2015).

Based on differences in energy intake, body weight gain, lipid profile, and mRNA level of genes involved in hepatic lipid and cholesterol metabolism, we can assume that protein from different dietary sources has a very significant effect on the lipid metabolism and metabolic function. Beef protein significantly increased the triglycerides accumulation and lipogenesis in liver but reduced the relative abundance of gut bacteria which negatively correlates with obesity.

## Conclusion

Intake of protein diet from beef, casein, and soy had similar effects on the lipid metabolism and triglycerides accumulation in mice when consumed with LFD. However, when the same proteins were consumed with high fat, distinctive effects were observed as beef protein diet increased the energy intake, enhanced serum, and liver markers associated with dyslipidemia, cholesterolemia, and atherosclerosis, which subsequently resulted in impaired glucose metabolism and insulin resistance, and higher weight gain. A comparison of the gut microbiome between lean and obese mice showed that all protein groups in HFD groups increased the *Firmicutes* to *Bacteriodetes* ratio, whereas beef protein with HFD greatly decreased the abundance of *Akkermansia* and other genera reported to have a negative correlation with obesity.

## Accession Information for Sequencing

Sequence data for the colonic microbiota has been uploaded in Sequence Read Archive of NCBI under accession code: SRP149955.

## Author Contributions

CL and MI designed the research. MI and CL wrote the manuscript. MI, MA, XZ, MH, and MZ acquired all the raw data. GZ and XX analyzed the data and criticized the manuscript. All authors reviewed the manuscript.

## Conflict of Interest Statement

The authors declare that the research was conducted in the absence of any commercial or financial relationships that could be construed as a potential conflict of interest.

## Supplementary Material

FIGURE S1 Rarefaction curves of colonic microbiota in all samples. Note: each line represents one sample.

TABLE S1 Dietary Composition for Low Fat Diets (D1250J) and High Fat Diets (D12492).

TABLE S2 Primers for real time PCR analysis of gene expression.

TABLE S3 Diversity estimation of colonic microbiota in all low-fat samples and high-fat samples.
