# Supplementary figures and images for: Beef, Casein, and Soy Proteins Differentially Affect Lipid Metabolism, Triglycerides Accumulation and Gut Microbiota of High-Fat Diet-Fed C57BL/6J Mice
Source: Front Microbiol. 2018 Sep 24;9:2200. doi: 10.3389/fmicb.2018.02200 (PMC6165900; doi:10.3389/fmicb.2018.02200)

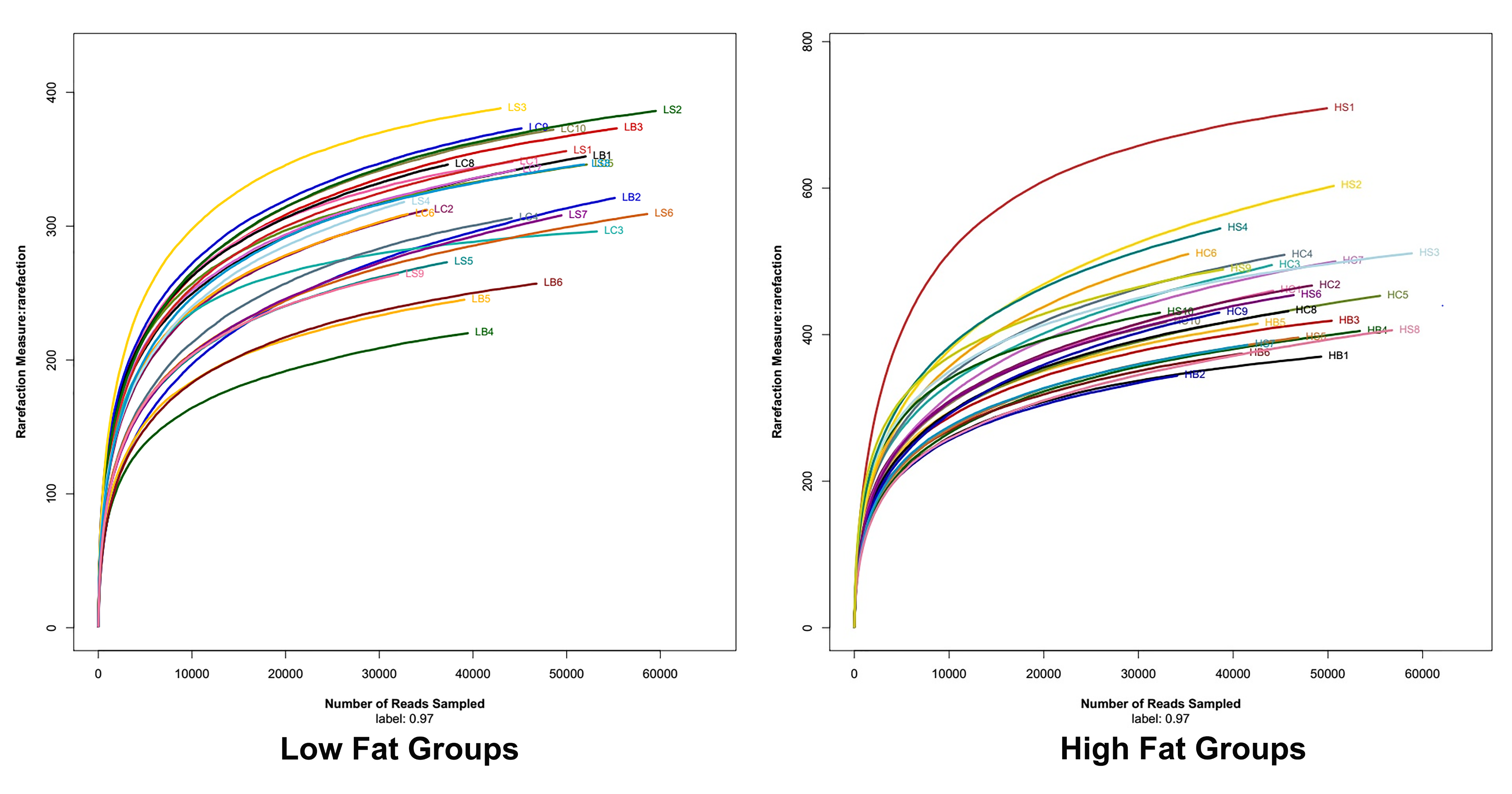

Supplement: FIGURE S1 — Rarefaction curves of colonic microbiota in all samples. Note: each line represents one sample. [file Image_1.TIFF]
