# Supplementary material for: Beef, Casein, and Soy Proteins Differentially Affect Lipid Metabolism, Triglycerides Accumulation and Gut Microbiota of High-Fat Diet-Fed C57BL/6J Mice
Source: Front Microbiol. 2018 Sep 24;9:2200. doi: 10.3389/fmicb.2018.02200 (PMC6165900; doi:10.3389/fmicb.2018.02200)
Supplement: TABLE S1 — Dietary Composition for Low Fat Diets (D1250J) and High Fat Diets (D12492). [file Table_1.DOCX]

**Table S1 Dietary Composition for Low Fat Diets (D1250J)**

| **Ingredients** | **Casein** | **Soy** | **Beef** | **Kcal** |
| --- | --- | --- | --- | --- |
|  | **g/kg** | **g/kg** | **g/kg** | **Kcal/kg** |
| **Protein Powder,80 Mesh** | 189.56 | 184.87 | 184.70 | 800 |
| **L-Cystine** | 2.843 | 2.843 | 2.843 | 12 |
| **Corn Starch** | 479.787 | 479.787 | 479.787 | 2025 |
| **Maltodextrin 10** | 118.47 | 118.47 | 118.47 | 500 |
| **Sucrose** | 65.21 | 65.21 | 65.21 | 275 |
| **Cellulose,BW200** | 47.39 | 47.39 | 47.39 | 0 |
| **Soybean oil** | 23.695 | 23.695 | 23.695 | 225 |
| **Lard** | 18.956 | 18.956 | 18.956 | 180 |
| **Mineral Mix S10026** | 11.308 | 8.67 | 10.84 | 0 |
| **DiCalcium Phosphate** | 12.819 | 15.25 | 18.06 | 0 |
| **Calcium Carbonate** | 9.85 | 8.20 | 6.00 | 0 |
| **Potassium Citrate, 1H2O** | 21.2 | 21.14 | 18.95 | 0 |
| **Vitamin Mix V10001** | 9.47 | 9.471 | 9.47 | 40 |
| **Choline Bitartrate** | 1.895 | 1.895 | 1.895 | 0 |
| **FD&C Yellow Dye #5** | 0.047 | 0.047 | 0.047 | 0 |
| **FD&C Red Dye #40** | 0 | 0 | 0 | 0 |
| **FD&C Blue Dye #1** | 0 | 0 | 0 | 0 |
| **Total** | 1012.557815 | 1005.929248 | 1006.366693 | 4057 |

**Table S1 Dietary Composition for High Fat Diets (D12492)**

| **Ingredients** | **Casein** | **Soy** | **Beef** | **Kcal** |
| --- | --- | --- | --- | --- |
|  | **g/kg** | **g/kg** | **g/kg** | **Kcal/kg** |
| **Protein Powder,80 Mesh** | 258.44 | 252.05 | 251.81 | 800 |
| **L-Cystine** | 3.876 | 3.876 | 3.876 | 12 |
| **Corn Starch** | 0 | 0 | 0 | 0 |
| **Maltodextrin 10** | 161.53 | 161.53 | 161.53 | 500 |
| **Sucrose** | 88.90 | 88.90 | 88.90 | 275 |
| **Cellulose,BW200** | 64.61 | 64.61 | 64.61 | 0 |
| **Soybean oil** | 32.30 | 32.30 | 32.30 | 225 |
| **Lard** | 316.59 | 316.59 | 316.59 | 2205 |
| **Mineral Mix S10026** | 10.94 | 8.56 | 10.32 | 0 |
| **DiCalcium Phosphate** | 9.076 | 12.39 | 16.22 | 0 |
| **Calcium Carbonate** | 12.55 | 10.29 | 7.30 | 0 |
| **Potassium Citrate, 1H2O** | 21.16 | 21.05 | 18.07 | 0 |
| **Vitamin Mix V10001** | 12.92 | 12.92 | 12.92 | 40 |
| **Choline Bitartrate** | 2.58 | 2.58 | 2.58 | 0 |
| **FD&C Yellow Dye #5** | 0 | 0 | 0 | 0 |
| **FD&C Red Dye #40** | 0 | 0 | 0 | 0 |
| **FD&C Blue Dye #1** | 0.064 | 0.064 | 0.064 | 0 |
| **Total** | 995.58 | 987.76 | 987.14 | 4057 |

**Supplementary Table S2. Primers for real time PCR analysis of gene expression**

| **Name** | **Forward Sequence** | **Reverse Sequence** |
| --- | --- | --- |
| *Gapdh* | AGGTCGGTGTGAACGGATTTG | TGTAGACCATGTAGTTGAGGTCA |
| *Scd - 1* | TTCTTACACGACCACCACCA | CCGAAGAGGCAGGTGTAGAG |
| *Fas* | AGAGATCCCGAGACGCTTCT | GCCTGGTAGGCATTCTGTAGT |
| *Acc - 1* | GCCTCTTCCTGACAAACGAG | TGACTGCCGAAACATCTCTG |
| *Srebp -1c* | CCCTGTGTGTACTGGCCTTT | TTGCGATGTCTCCAGAAGTG |
| *Srebpf2* | CCAAAGAAGGAGAGAGGCGG | CGCCAGACTTGTGCATCTTG |
| *Hmgcr* | AGGCCATGCATCCGGAAAA | GAGCCCCATGCATGCTAAGT |
| *Mgat1* | TGGTGCCAGTTTGGTTCCAG | TGCTCTGAGGTCGGGTTCA |
| *Fabp4* | AAGGTGAAGAGCATCATAACCC | TCACGCCTTTCATAACACATTCC |
